# The role of precentral cerebellar vein in neurosurgery: An in-depth review of anatomy, implications, and risks

**DOI:** 10.1016/j.bas.2024.102861

**Published:** 2024-07-06

**Authors:** Oday Atallah, Gianluca Scalia, Mohammed A. Azab, Ahmed Muthana, Omar Wawi, Vivek Sanker, Almutasimbellah K. Etaiwi, Samer S. Hoz

**Affiliations:** a Department of Neurosurgery, Hannover Medical School, Hannover, Germany; b Neurosurgery Unit, Department of Head and Neck Surgery, ARNAS Garibaldi, Catania, Italy; c Departemnt of Neurosurgery, Cairo University Hospital, Cairo, Egypt; d College of Medicine, University of Baghdad, Baghdad, Iraq; e Department of Radiology, Sana Hospital Offenbach, Offenbach, Germany; f Department of Neurosurgery, Trivandrum Medical College, Kerala, India; g Department of Neurosurgery, Saudi German Hospital Ajman, United Arab Emirates; h Department of Neurosurgery, University of Pittsburgh Medical Center (UPMC), 1542 Spring Park Walk, Pittsburgh, 15064, Pennsylvania, United States

**Keywords:** Precentral cerebellar vein, Neurosurgery, Surgical anatomy, Neurosurgical implications, Posterior fossa tumors

## Abstract

**Background:**

The Precentral Cerebellar Vein (PCV) plays a crucial role as an anatomical landmark in neurosurgery, and the possibility of its safe sacrifice is controversial. Understanding its anatomical nuances and clinical implications is fundamental in enhancing neurosurgical practice.

**Methods:**

A systematic review following PRISMA guidelines was conducted to consolidate literature on the PCV. PubMed, Scopus, and Web of Science were systematically searched using predefined criteria. Studies providing complete research texts in English, focusing on the PCV's surgical anatomy and neurosurgical implications were included.

**Results:**

Fourteen articles met inclusion criteria, exploring the PCV's anatomical variations, trajectory, dimensions, and connections. The PCV's utility in localizing posterior fossa tumors was underscored, aiding in surgical precision. However, sacrifices of the PCV or minor veins for access to quadrigeminal areas posed postoperative risks, emphasizing the need for careful preoperative planning. Additionally, the PCV's diagnostic value in venous malformations and developmental anomalies was highlighted.

**Conclusions:**

This comprehensive review accentuates the pivotal role of the PCV in neurosurgery. While serving as a vital guide in procedures, it poses potential risks when manipulated. Understanding its multifaceted significance, from anatomy to clinical implications, is paramount for informed decision-making and minimizing complications in neurosurgical interventions.

## Highlights

• The PCV plays a crucial role as a landmark in microsurgical procedures, helping neurosurgeons precisely localize tumors in the posterior fossa.
• The PCV's clinical value in diagnosing venous malformations and developmental anomalies.
• Venous infarction and hemorrhage are example of the potential complications that may arise from PCV manipulation during surgery.

## 1 Introduction

The Precentral Cerebellar Vein (PCV) resides within the fissure separating the lingula and the central lobule of the cerebellum. It extends upwards and forwards behind the anterior medullary velum of the 4th ventricle and is alternatively referred to as the vein of the cerebellomesencephalic fissure. The course of the PCV runs superiorly within the quadrigeminal cistern, situated behind the tectal plate, originating at the convergence point of the brachial veins above the middle cerebellar peduncle. As a crucial anatomical landmark, the PCV aids in identifying the dorsal midbrain and the superior cerebellar vermis. Additionally, it serves a significant drainage function, contributing to the great vein of Galen (GVG) and occasionally joins the superior vermian vein during its ascent to form the superior cerebellar vein. Both pathways ultimately join the GVG (Berker et al., 2023).

The primary objective of this systematic review is to consolidate and analyze existing literature on the PCV, particularly focusing on its surgical anatomy and neurosurgical implications. This critical analysis aims to elucidate the significance of the PCV in neurosurgical procedures and its potential impact on clinical practice.

## 2 Methods

This review comprehensively examined relevant literature concerning the Precentral Cerebellar Vein (PCV). To ensure a systematic and comprehensive assessment, researchers conducted a systematic review following the guidelines outlined in the PRISMA (Preferred Reporting Items for Systematic Reviews and Meta-Analyses) framework (Mizukami et al., 1969). PubMed, Scopus, and Web of Science databases were systematically searched to gather pertinent resources. Articles not available in full text, animal studies, and those published in languages other than English were excluded from the analysis. Inclusion criteria were strictly based on studies that provided complete research text. The search terms "precentral cerebellar vein," "vein of the cerebellomesencephalic fissure," and "PCV neurosurgical applications' ' were used to identify relevant literature.

**Fig. 1:**
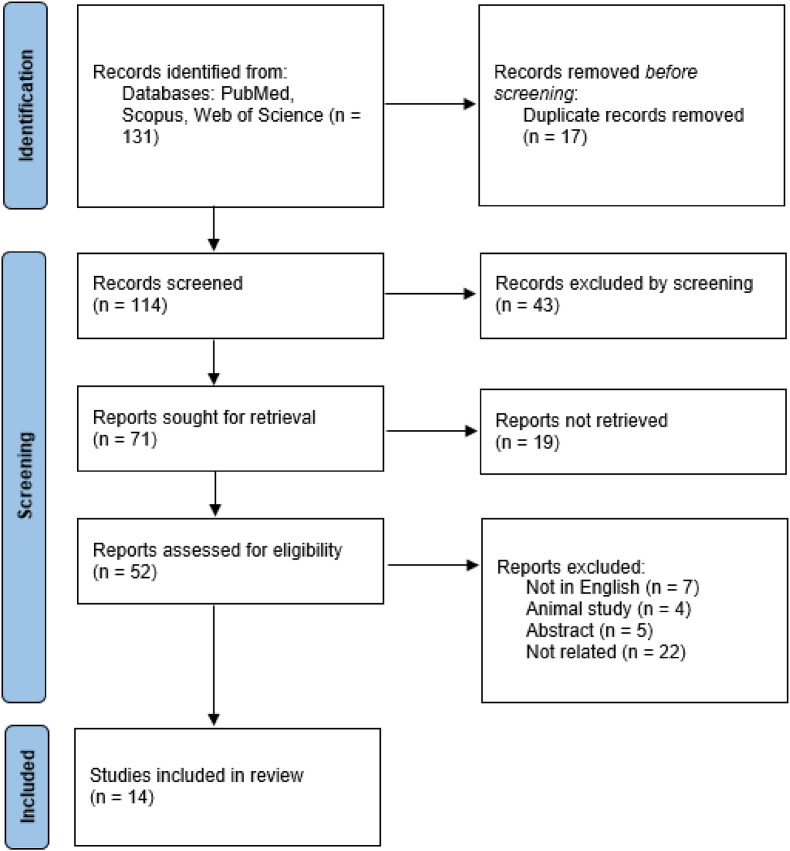
Flow diagram of the related articles.

The initial screening involved a review of paper titles and abstracts to select appropriate articles while removing duplicate entries. Subsequently, a dual approach was employed where two authors independently conducted an in-depth study. Any discrepancies or inconsistencies between the two reviewers were resolved through consultation with a third reviewer, ensuring a definitive analysis (Fig. 1). The collected data from these studies focused on the surgical anatomy and neurosurgical implications associated with the PCV.

## 3 Results

According to our predefined inclusion and exclusion criteria, a total of 14 articles were identified that addressed anatomical variations and neurosurgical implications associated with the Precentral Cerebellar Vein (PCV) by reviewing existing literature and original research. These articles collectively explored various facets of the vein's surgical anatomy, encompassing its origin, trajectory, diameter, branching pattern, and its connections to the cisterna quadrigeminalis and the colliculo-central angle. Furthermore, these studies examined the practical application of the PCV in neurosurgical procedures Table 1. Notably, its significance in localizing tumors within the posterior fossa was highlighted.

**Table 1:** Summary of the available studies involving the PCV.

	Study type	Pathology	Origin of the vein	Trajectory of the vein	Drainage	Diameter	Cisterna	Displacement pattern	Clinical Significance
**Hopkins LN 1975** (Hopkins et al., 1975)	Case report	Vermian cerebellar astrocytomas	Within the precentral cerebellar fissure	NM	NM	NM	Quadrigeminal cistern at the colliculocentral point	In all 3 cases of cerebellar astrocytomas, the vein was displaced forward and upward.	Correlation between the displacement pattern and tumor location is possible
**Mizukami 1970** (Mizukami et al., 1969)	Case series	Cerebellar hemispheric tumors	NM	NM	NM	NM	NM	Forward displacement	Correlation between the displacement pattern and tumor location is possible
		4th Ventricular tumors	NM	NM	NM	NM	NM	Backward displacement	
		Pontine tumors	NM	NM	NM	NM	NM	Backward displacement	
		Pineal tumors	NM	NM	NM	NM	NM	Downwards, and backwards displacement	
**Crevits et al., 1978** (Crevits et al., 1978)	Case series	vascular malformation	NM	NM	NM	NM	Superior cerebellar cisterna	Not applicable	CPV may be a major component of vascular malformation
**Kodera et al., 2011** (Kodera et al., 2011)	Case series	Pineal body tumors	NM	NM	NM	NM	NM	Not applicable	PCV should be divided as distally as possible from the vein of Galen as possible
**Mario Giordano 2009** (Giordano et al., 2009)	Case series	Different pathologies	Arising deep in cerebellomesencephalic fissure	NM	Drains to: vein of Galen or via the superior vermian vein, to form the superior cerebellar vein (drains to vein of Galen	NM	NM	Not applicable	Possibility of depicting the PCV using 3-dimensional neuronavigation with an accuracy comparable to that of digital subtraction angiography.
**Markus E. Krogager 2019** (Krogager et al., 2021)	Survey	Not applicable	NM	NM	NM	NM	NM	Not applicable	Five of 29 surgeons would never occlude this vein, in 13 cases of PCV occlusion, no effect, while one case was reported to have infarction
**Kieffer SA 1975** (Kieffer et al., 1975)	Case series	CPA tumors	NM	NM	NM	NM	NM	Upward and posterior displacement	Buckling back of the PCV indicates upward growth of CPA tumor
**Chaynes, 2003** (Chaynes, 2003)	Cadaveric study	Not applicable	Union of two brachial tributaries located over the med. cerebel.ped	NM	Drains to: vein of Galen in 4/25 specimens, fused with the superiorvermian vein in 21/25, as the superiorcerebellar vein drained into the vein of Galen	0.8–1.9 mm	NM	NM	To avoid intraoperative venous infarction, it is important to use angiography to determine the venous organization before surgery and to estimate the permeability and size of the branches of the deep venous system
**Kilic 2005** (Kılıç et al., 2005)	Cadaveric sudy	NM	Union of bilateral superior. cerebellar. peduncular veins	NM	Drains to: vein of Galen (8/10) and into straight sinus (2/10)	NM	NM	NM	Anatomically, the PCV is highly variable. New and more sophisticated vascular imaging technology is advocated
**Rhoton, 2000** (Rhoton, 2000)	Cadaveric study	NM	Union of bilateral superior. cerebellar. peduncular. veins	NM	Drains to: vein of Galen or through the superior vermian vein into vein of Galen.	NM	Quadrigeminal cistern	NM	The importance of preserving the bridging veins in posterior fossa surgery should be considered
**Suzuki et al., 2005** (Suzuki et al., 2005)	Case series	NM	NM	NM	Drains to: vein of Galen	NM	NM	NM	In occipital transtentoril approach, the PCV could be sectioned during tumor removal. Proper preoperative identification of the anatomical variations, course and drainage points should be considered.
**Matsushima et al., 1983** (Matsushima et al., 1983)	Cadaveric study	Below colliculi from the union of the veins of the superior cerebellar peduncle	NM	NM	Vein of Galen or via the superior vermian vein	1–1.9 mm	Quadrigeminal cistern	NM	The importance of preserving the bridging veins in posterior fossa surgery should be considered
**Huang and Wolf 1965** (Huang et al., 1966)	Radiologic study	Precentral cerebellar vein often fed by a sup. cerebel. hemisph. trib (the preculminate vein	NM	NM	Drains to: vein of Galen	NM	NM	NM	PCV could be absent or replaced by veins laterally that join vein of Galen
**Charles Lee 1996** (Lee et al., 1996)	Case series	Developmental venous anomalies	NM	NM	NM	NM	NM	NM	The PCV could be one of the deep draining veins of venous anomalies.

## 4 Discussion

The Precentral Cerebellar Vein (PCV), located within the posterior cerebellar fossa, exhibits considerable anatomical variability. Attributes such as its origin, trajectory, branching patterns, dimensions, depth, proximity to the clivus, and interconnections with adjacent structures are essential considerations in discerning its diverse forms. The PCV is positioned between the cerebellar central lobule and the lingula, originating within the precentral cerebellar fissure (Hopkins et al., 1975). This vein can manifest as paired vessels or a network of smaller veins running parallel to the roof of the fourth ventricle within the precentral cerebellar fissure, eventually converging to form a unified central trunk within the fissure (Giordano et al., 2009; Krogager et al., 2022). In 72% of cases studied, a single trunk was observed in the PCV, while in 12% of cases, PCVs were completely independent, and in 16% of cases, no discernible vein was present (Legiehn et al., 2008). The width of the vein ranged from 0.8 to 1.9 mm (Kieffer et al., 1975).

**Fig. 2:**
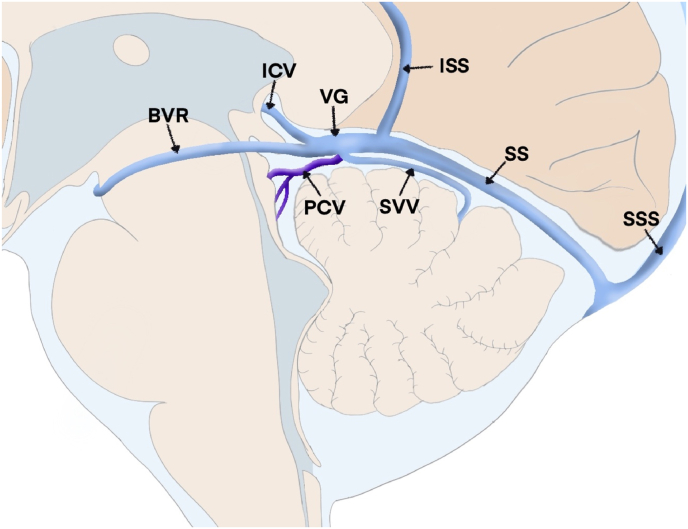
An illustration about the PCV.

Upon emergence from the precentral cerebellar fissure, the common trunk of the PCV ascends into the quadrigeminal cistern, entering at the colliculo-central point situated midway between the inferior colliculi inferiorly and the central lobule superiorly(Giordano et al., 2009). Subsequently, it progresses anteriorly within the cisterna ambiens, connecting either to the Great Vein of Galen (GVG) or the posterior segment of the internal cerebral vein (Kodera et al., 2011) (Fig. 2). The PCV delineates three distinct segments along its path: the fissural segment, the anterior culmine segment, and the cisternal segment. Occasionally, an additional parenchymal segment might precede the fissural segment in angiograms (Kieffer et al., 1975). The initial part of the PCV follows a straight trajectory behind the apex of the fourth ventricle's roof and the base of the aqueduct (Hopkins et al., 1975). The subsequent segment initiates a directional shift approximately 3–4 mm away from the fourth ventricle, adopting a more oblique upward path (Kieffer et al., 1975). The third segment commences as the vein departs from the vermis and enters the GVG (Kieffer et al., 1975). The brachial veins, originating over the brachium pontis, traverse medially across the brachium conjunctivum and the lingula before converging at the fissure between the lingula and the central lobule of the cerebellum. From this convergence point, a singular central trunk, termed the PCV, ascends behind the anterior medullary velum (Kodera et al., 2011). The junction of the brachial veins or the PCV's origin can vary from a lower position within the fissure to a higher location in the quadrigeminal cistern (Kieffer et al., 1975). While typically the PCV shows as a midline single vessel, examinations revealed bilaterally present PCVs in 30% of specimens (Kanno, 1995). The PCV's drainage into the GVG occurs through two routes: direct entry into the GVG at its junction with the straight sinus, or the merging of the superior vermian vein with the PCV to form the superior cerebellar vein (Giordano et al., 2009). The GVG typically intersects the straight sinus perpendicularly, establishing retrograde blood flow from the inferior sagittal sinus to the straight sinus. The angle formed by the GVG and the straight sinus averages at 67.1 ± 31.99° (Lee et al., 1996; Peeters, 1973). Studies observed that in 86.4% of cases, the PCV drained into the GVG, while in 13.6% of cases, the PCV connected to both sides of the internal cerebral vein (Matsushima et al., 1983). Notably, variations were observed wherein the brachial veins occasionally flowed independently into the GVG rather than merging (Matsushima et al., 1983). Moreover, the PCV was found to amalgamate with smaller veins from the vermis' anterior surface and the adjacent cerebellar hemisphere at the junction between the inferior colliculi and the anterior superior edge of the cerebellum (Matsushima et al., 1983).

The average distance from the PCV to the back of the clivus, across a sample of 40 healthy adults, measured 39 mm, ranging from 36 to 43 mm. Children displayed a shorter distance between these points due to their smaller head sizes (Kodera et al., 2011; Krogager et al., 2022).

Historically, cerebral angiography posed challenges in initially diagnosing posterior fossa tumors. However, evaluating the PCV facilitated better assessment of the superior vermis, the aqueduct, the upper portion of the fourth ventricle, and the midbrain. Assessment of the PCV with other arteries like the precentral cerebellar artery increased the diagnostic utility of angiography for posterior fossa tumors (Kodera et al., 2011). Yet, the variability of anatomical structures can limit surgical exposure, compelling surgeons to consider sacrificing veins like the PCV or other minor veins for accessing quadrigeminal areas. However, such venous sacrifice might lead to postoperative complications, including cerebral edema or cerebellar swelling, emphasizing the necessity for meticulous preoperative planning to identify those veins (Matsushima et al., 1983). Despite employing advanced techniques, proper identification of small veins is still difficult, underscoring the requirement for further advancements in noninvasive imaging technology (Giordano et al., 2009).

Astrocytomas are among the most common cerebellar tumors (Legiehn et al., 2008). Their lateral view typically reveals the PCV's path within the lateral recess of the fourth ventricle, alongside a displacement of the inferior vermian vein toward the opposite side. Such displacements indicate signs of transtentorial herniation (Legiehn et al., 2008). Posterior fossa tumors often cause an enlargement of the normal convexity of the PCV in the anterior and superior directions (Kodera et al., 2011).

Distinct venous signs indicative of fourth ventricle tumors that involve tonsil displacement include a posterior shift of the venous copular point, closure of the venous tonsillovermian angle, and stretching of tributaries of the vein in the lateral recess of the fourth ventricle, visually shortened on lateral projection (Legiehn et al., 2008). The PCV shifts backward within a range of 38–56 mm from the clivus to the colliculo-central point due to cerebellar tumors affecting the inferior vermis and tonsil (Kodera et al., 2011).

Pineal body tumors can cause the PCV to develop a convex shape with a subtle posterior curvature, becoming more pronounced with tumor growth. This leads to a downward and backward movement of the colliculo-central point (Kodera et al., 2011).

The infratentorial-supracerebellar and occipital-transtentorial routes are commonly employed to access pineal region tumors. For centrally located tumors extending into the posterior incisural space below the collicular plate and displacing the cerebellum, an infratentorial-supracerebellar approach is preferred (Berker et al., 2023). However, this approach often results in extensive coagulation and ablation of the PCV, causing thrombosis in the veins of Rosenthal and the internal cerebral vein, potentially leading to brain damage and edema (Huang et al., 1966; Krogager et al., 2021).

Sacrificing the bridging veins is considered safe by some neurosurgeons in order to easily obtain a generous surgical exposure and reduce the risk of uncontrolled bleeding. However, the results of sacrificing these veins are unpredictable (Peeters, 1973). Moreover, several case reports have challenged the concept of deliberate sacrifice of cerebellar bridging veins given the occasional complications associated (Jakola et al., 2013; Koh and Park, 2017; Krogager et al., 2021). Kodera et al. reported that they usually preserve the PCV, and in cases of utmost need to sacrifice this vein, they recommend to divide it away from the vein of gallen to avoid thrombosis (Kodera et al., 2011). It is common to sacrifice the PCV without clinical complications, however, Kanno et al. reported that thrombosis of the basal veins of Rosenthal and cerebellar infection resulted from coagulating the PCV very close to the confluence of basal veins. Hence, great attention should be taken when coagulating the PCV and it should be done so far from the joint point of the basal veins as thrombosis may extend into the deep venous system (Kanno, 1995). Considerable knowledge of the anatomical variations of the PCV nd branching features is of great importance to avoid catastrophic postoperative complications. Different venous imaging modalities could be useful for preoperative assessment of these venous networks. Generally, iatrogenic venous damage could cause several problems ranging from edema to fatal hemorrhagic infarctions (Koh and Park, 2017). In posterior fossa surgery, protection of the bridging veins between the cerebellar surface and the tentorium is considerably essential. A substantial surgical effort should be considered to protect the bridging veins as much as possible. In general, when dissecting a bridging vein, the vein has to be freed at a length of 10–20 mm from the arachnoid and cortex (Berker et al., 2023). In supracerebellar infratentorial approaches, dynamic retraction should be applied to avoid compressive injury to the bridging veins. Sacrificing a bridging vein should be the last option, while using different techniques to protect the vein as much as possible should be tried initially. Temporary clipping of the venous wall is a possible way to avoid venous division. Gentle hemostasis using surgecele or other non thrombotic hemostatic materials would be suitable for small punctures avoiding venous division. In all situations, the venous bridging veins should be respected as much as possible.

Venous malformations, the most common type of vascular malformations, typically cause symptoms related to pressure or compromised blood circulation (Lee et al., 1996). Diagnosis of posterior fossa venous malformations is facilitated by evaluating the PCV, which may show abnormal drainage patterns indicating venous malformations (Crevits et al., 1978).

Developmental Venous Anomalies (DVAs) are vascular malformations characterized by a central draining vein surrounded by a network of dilated veins resembling a "caput medusae." In the infratentorial compartment, the PCV serves as a conduit for venous outflow from the dilated veins, distinguishing DVAs from other vascular malformations (Lee et al., 1996; Legiehn et al., 2008).

Vein of Galen malformations are brain vascular defects causing vein enlargement and aneurysmal growth. Diagnosis typically involves angiography to detect dilated GVG as a tumor-like growth. PCV dilation due to increased blood flow serves as a diagnostic indicator of Vein of Galen malformations (Peeters, 1973).

Embolization is a treatment method to reduce abnormal blood flow in venous malformations, including Vein of Galen malformations. Inserting embolic agents into the PCV helps decrease blood flow and pressure within the malformation, yet it can lead to PCV thrombosis, causing brain damage and edema (Peeters, 1973).

## 5 Conclusion

This review extensively explores the intricate details of the Precentral Cerebellar Vein (PCV) and its clinical significance in neurosurgery. It highlights how crucial the vein is as an important structure to identify and protect during supracerebellar infratentorial approaches. Moreover, the review shows how the PCV could be involved in the surgical and interventional procedures for posterior fossa vascular malformations. However, it also highlights the potential risks involved in manipulating the PCV during surgery, such as blood clotting issues and bleeding. Understanding these aspects is crucial for surgeons to make informed decisions and reduce the chances of complications. This comprehensive overview highlights the PCV's significance in neurosurgery, from its anatomy to its practical uses and the associated risks involved in its manipulation during surgical procedures.

## Conflict of interest and financial support

The authors have no Conflict of interest and financial support to disclose.

## Declaration of competing interest

Oday Atallah: none.

Gianluca Scalia: none.

Mohammed A. Azab: none.

Ahmed Muthana: none.

Omar Wawi: none.

Almutasimbellah K. Etaiwi: none.

Vivek Sanker: none.

Samer S. Hoz: none.

## Glossary

DVA: Developmental Venous Anomalies
GVG: Vein of Galen
PCV: Precentral Cerebellar Vein
